# Improving management of neonatal infections

**DOI:** 10.1016/S0140-6736(18)31432-6

**Published:** 2018-07-06

**Authors:** Anna C Seale, Ramesh Agarwal

**Affiliations:** Epidemiology and Population Health, London School of Hygiene and Tropical Medicine, London WC1E 7HT, UK (ACS); College of Health and Medical Sciences, Haramaya University, Haramaya, Ethiopia (ACS); KEMRI-Wellcome Trust Research Programme, Kilifi, Kenya (ACS); and Newborn Health Knowledge Centre, WHO Collaborating Centre for Training and Research in Newborn Care, Neonatal Division at the Department of Paediatrics, All India Institute of Medical Sciences, New Delhi, India (RA)

Infections causing sepsis, meningitis, or pneumonia contributed directly to around 0·6 million neonatal deaths worldwide in 2016,^1^ and indirectly to many more through pathways leading to preterm birth and neonatal encephalopathy. Despite this knowledge, understanding of the causes of neonatal infection, particularly in resource-poor settings, is limited. Treatment in these settings usually relies on the sensitive but non-specific clinical diagnosis of possible serious bacterial infection (pSBI),^2^ made by front-line health-care workers and defined according to set criteria. Of the almost 7 million neonates needing treatment worldwide each year based on this diagnosis,^3^ most are not tested for specific infectious causes and many are likely to have non-infectious conditions (figure).

In *The Lancet*, Samir Saha and colleagues report the Aetiology of Neonatal Infection in South Asia (ANISA) study,^4^ which is an important step forward in understanding the infectious causes of neonatal pSBI. The community-based study design is an advance on previous studies, which have been mostly facility-based, and often performed limited microbiological investigations. ANISA enrolled 84 971 mothers antenatally across five sites in Bangladesh, India, and Pakistan, and used community health-care workers to follow up neonates after birth. Antenatal recruitment of mothers meant that neonates who died shortly after birth were counted and that pSBIs were quickly identified by community health-care worker follow-up. Systematic sampling and testing with conventional and molecular laboratory methods maximised pathogen detection. Reductions in specificity of diagnosis and identification of multiple organisms by molecular diagnostics were mitigated by use of control data and Bayesian partially latent class modelling to estimate attributable proportions for specific infectious causes.

Saha and colleagues’ findings for the causes of pSBIs and the non-specificity of this classification as a diagnosis are important. Of 6022 pSBI episodes, only 16% had attributed bacterial causes, and 102 (2%) of 4859 tested blood samples had clinically significant pathogens isolated by culture. More specific clinical algorithms and point-of-care diagnostics are needed to direct antibiotic treatment to those who need it, especially as antibiotic treatment for neonatal pSBI is scaled up; WHO guidelines recommend that when referral to hospital is not possible, antibiotic treatment should be given to outpatients to expand access to care.^5^ Of note, however, this recommendation was informed by pragmatic antibiotic trials that used pSBI as a clinical diagnosis and tested equivalence of regimens.^6–8^ The ANISA study findings add to concerns about the use of non-specific clinical diagnoses for such trials^9^ and underscore the uncertainty in their findings.

As well as what it found, ANISA is important for what it did not find. Among 71 361 livebirths, 3061 (4%) babies died by the end of follow-up, most of these soon after birth. Despite active follow-up by community health-care workers, only 689 (23%) babies who died were assessed by a physician before death, and only 349 (11%) had samples taken in the 7 days before death.^4^ Under-representation of deaths is a limitation in terms of attributing infectious causes, but showing how many neonates who die and who are not seen or investigated for infection is important. These data are often not captured, or are not reported, and the extent to which the sickest neonates in the community, in research or in clinical practice, are not seen is unknown in many resource-poor settings. Improving understanding of the causes of these deaths is crucial. Infection is likely to be an important direct and indirect contributor, as are preterm birth and neonatal encephalopathy. In ANISA, the number of attributed infections was nearly double that among babies who died than among those who survived, and more than 90% of the infectious causes in those who died were bacterial.^4^

Further development of the evidence base to better direct interventions towards the highest burden of neonatal mortality at and in the few days after birth will need new approaches. One such approach is post-mortem investigation with minimally invasive tissue sampling, which may be more acceptable than complete diagnostic autopsy and could allow investigation of stillbirths and neonates not seen or assessed before death.^10^ The Child Health and Mortality Prevention Surveillance study aims to use such techniques.^11^ Another potential approach is the use of maternal vaccines in the context of trials, and surveillance after implementation, to determine the contributions of specific infectious causes. Maternal vaccines are being developed for various pathogens, such as respiratory syncytial virus and group B streptococcus.^12^

The ANISA study has advanced understanding of neonatal infection and highlighted the limitations of current management strategies. Ways to address these issues must be urgently sought, and it must be remembered that the neonates not seen matter as much as those that are.

## Figures and Tables

**Figure:**
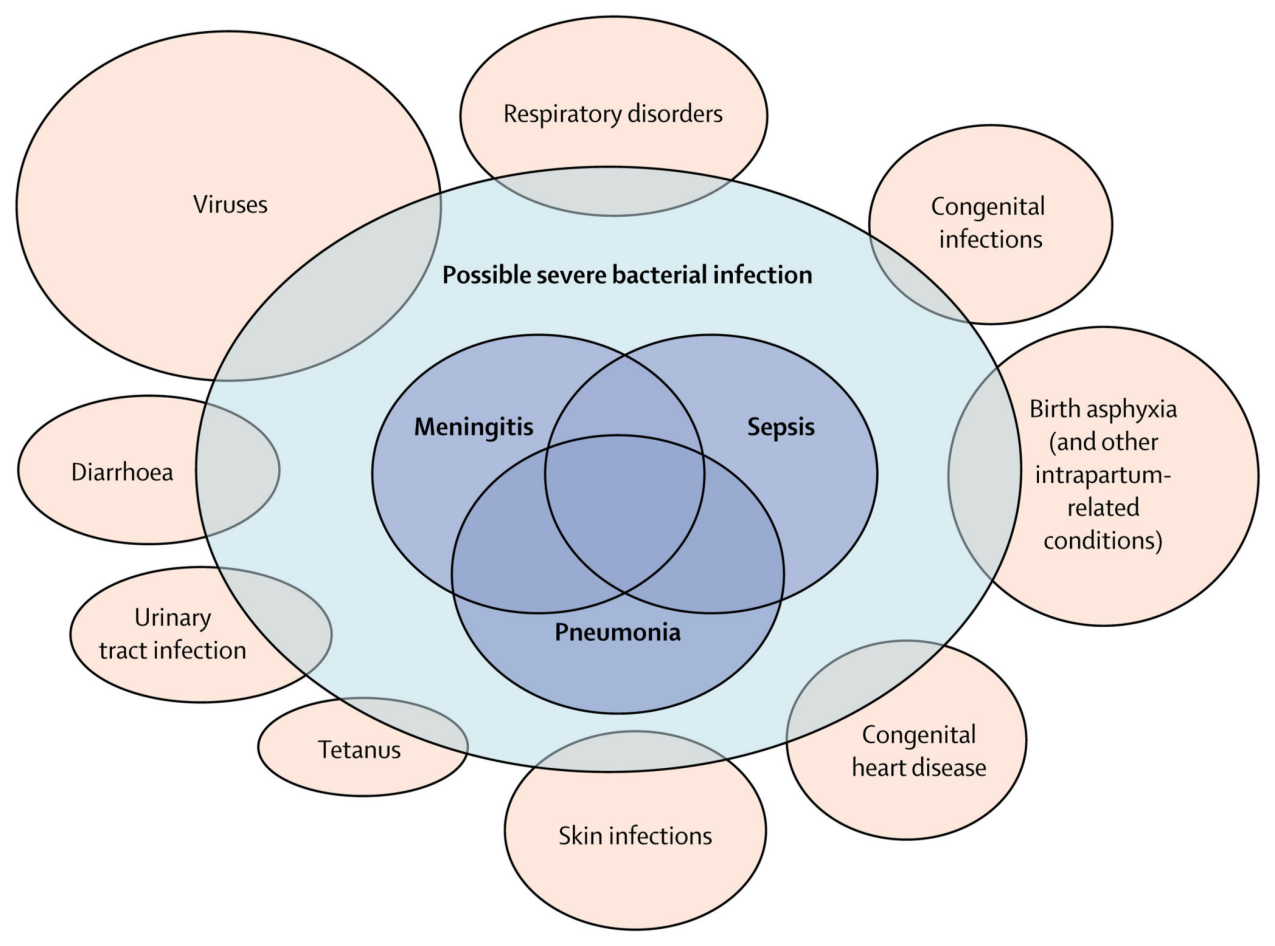
Overlap between possible serious bacterial infections and other clinical syndromes Reproduced from Seale et al.^3^

## References

[1] Liu L, Oza S, Hogan D (2016). Global, regional, and national causes of under-5 mortality in 2000-15: an updated systematic analysis with implications for the Sustainable Development Goals. Lancet.

[2] Young Infants Clinical Signs Study Group (2008). Clinical signs that predict severe illness in children under age 2 months: a multicentre study. Lancet.

[3] Seale AC, Blencowe H, Manu AA (2014). Estimates of possible severe bacterial infection in neonates in sub-Saharan Africa, south Asia, and Latin America for 2012: a systematic review and meta-analysis. Lancet Infect Dis.

[4] Saha SK, Schrag SJ, El Arifeen S (2018). Causes and incidence of community-acquired serious infections among young children in south Asia (ANISA): an observational cohort study. Lancet.

[5] WHO (2015). Guideline: managing possible serious bacterial infection in young infants when referral is not feasible.

[6] Baqui AH, Saha SK, Ahmed AS (2015). Safety and efficacy of alternative antibiotic regimens compared with 7 day injectable procaine benzylpenicillin and gentamicin for outpatient treatment of neonates and young infants with clinical signs of severe infection when referral is not possible: a randomised, open-label, equivalence trial. Lancet Glob Health.

[7] Mir F, Nisar I, Tikmani SS (2017). Simplified antibiotic regimens for treatment of clinical severe infection in the outpatient setting when referral is not possible for young infants in Pakistan (Simplified Antibiotic Therapy Trial [SATT]): a randomised, open-label, equivalence trial. Lancet Glob Health.

[8] Tshefu A, Lokangaka A, Ngaima S (2015). Simplified antibiotic regimens compared with injectable procaine benzylpenicillin plus gentamicin for treatment of neonates and young infants with clinical signs of possible serious bacterial infection when referral is not possible: a randomised, open-label, equivalence trial. Lancet.

[9] Mulholland K, Carlin JB, Duke T, Weber M (2014). The challenges of trials of antibiotics for pneumonia in low-income countries. Lancet Respir Med.

[10] Bassat Q, Castillo P, Martinez MJ (2017). Validity of a minimally invasive autopsy tool for cause of death determination in pediatric deaths in Mozambique: an observational study. PLoS Med.

[11] Farag TH, Koplan JP, Breiman RF (2017). Precisely tracking childhood death. Am J Trop Med Hyg.

[12] Sobanjo-Ter Meulen A, Abramson J, Mason E (2015). Path to impact: a report from the Bill and Melinda Gates Foundation convening on maternal immunization in resource-limited settings; Berlin—January 29–30, 2015. Vaccine.

